# Control of the Inflammatory Macrophage Transcriptional Signature by miR-155

**DOI:** 10.1371/journal.pone.0159724

**Published:** 2016-07-22

**Authors:** Kyle A. Jablonski, Andrew D. Gaudet, Stephanie A. Amici, Phillip G. Popovich, Mireia Guerau-de-Arellano

**Affiliations:** 1 School of Health and Rehabilitation Sciences, Medical Laboratory Science Division, The Ohio State University, Columbus, Ohio, United States of America; 2 Department of Neuroscience, Wexner Medical Center at The Ohio State University, Columbus, Ohio, United States of America; 3 Department of Microbial Infection and Immunity, Wexner Medical Center at The Ohio State University, Columbus, Ohio, United States of America; Universitatsklinikum Freiburg, GERMANY

## Abstract

Inflammatory M1 spectrum macrophages protect from infection but can cause inflammatory disease and tissue damage, whereas alternatively activated/M2 spectrum macrophages reduce inflammation and promote tissue repair. Modulation of macrophage phenotype may be therapeutically beneficial and requires further understanding of the molecular programs that control macrophage differentiation. A potential mechanism by which macrophages differentiate may be through microRNA (miRNA), which bind to messenger RNA and post-transcriptionally modify gene expression, cell phenotype and function. We hypothesized that the inflammation-associated miRNA, miR-155, would be required for typical development of macrophage inflammatory state. miR-155 was rapidly up-regulated over 100-fold in inflammatory M1(LPS + IFN-γ), but not M2(IL-4), macrophages. Inflammatory genes *Inos*, *Il1b* and *Tnfa* and their corresponding protein or enzymatic products were reduced up to 72% in miR-155 knockout mouse M1(LPS + IFN-γ) macrophages, but miR-155 deficiency did not affect expression of the M2-associated gene *Arg1* in M2(IL-4) macrophages. Additionally, a miR-155 oligonucleotide inhibitor efficiently suppressed *Inos* and *Tnfa* gene expression in wild-type M1(LPS + IFN-γ) macrophages. Comparative transcriptional profiling of unstimulated and M1(LPS + IFN-γ) macrophages derived from wild-type (WT) and miR-155 knockout (KO) mice revealed that half (approximately 650 genes) of the signature we previously identified in WT M1(LPS + IFN-γ) macrophages was dependent on miR-155. Real-Time PCR of independent datasets confirmed that miR-155 contributed to suppression of its validated mRNA targets *Inpp5d*, *Tspan14*, *Ptprj* and *Mafb* and induction of *Inos*, *Il1b*, *Tnfa*, *Il6* and *Il12*. Overall, these data indicate that miR-155 plays an essential role in driving the inflammatory phenotype of M1(LPS+ IFN-γ) macrophages.

## 1. Introduction

Macrophages are found in all tissues of the body and help maintain homeostasis during embryonic development and throughout life [1,2]. In response to inflammatory stimuli (e.g., infection or tissue injury), resident macrophages become activated and blood monocytes are recruited to the inflammatory focus where they differentiate into macrophages. Collectively, the functions of resident and recruited macrophages are essential for ensuring tissue sterility and restoring homeostasis through the induction of wound healing and repair [3,4].

*In vivo*, cues in the tissue microenvironment, including cytokines and/or pathogen-or danger-associated molecular patterns (PAMPs or DAMPS, respectively) from pathogens or dying/damaged cells promote inflammatory macrophage phenotype and function [3,5]. Evidence for the existence of a spectrum or wheel of plastic macrophage phenotypes *in vivo* has been gathered in recent years [6,7]. Such varied phenotypes would be required to adapt to different and/or overlapping environmental stimuli and *in vivo* roles. *In vitro*, discrete macrophage phenotype models have been created to model some of these phenotypes [8]. For example, macrophages stimulated with interferon-γ (IFN-γ) and TLR agonists (e.g., lipopolysaccharide (LPS)) differentiate into one of the “classically” activated inflammatory M1 spectrum macrophages [8–12], further defined by the recently proposed nomenclature as M1(LPS + IFN-γ) macrophages [10]. Other M1 spectrum macrophages include M(LPS) and M(IFN-γ) stimulated macrophages [9,10]. Conversely, stimulating macrophages with anti-inflammatory cytokines like IL-4 or IL-13 has been described to generate alternatively activated, M2 or M(IL-4) macrophages [13–15]. *In vivo*, these cytokines are produced by T helper 2 (Th2) lymphocytes during immune responses to parasitic infections or allergens.

Murine inflammatory M1(LPS + IFN-γ) macrophages express cytokines such as TNF-α, IL-1β, IL-6 and IL-12, chemokines such as CCL5 and CXCL8 and surface molecules such as CD38, CD80 and CD86 [16,17]. They also express the enzyme inducible nitric oxide synthase (iNOS) which transforms arginine into the oxidizing and microbicidal product nitric oxide (NO) for resistance to bacterial infection [3,8–10,12,18]. However, excess or unresolved inflammatory macrophage responses can cause chronic inflammation and tissue damage. Indeed, inflammatory macrophages have been implicated in the pathogenesis of several inflammatory conditions including atherosclerosis, diabetes and glomerulonephritis [8,19,20]. In the nervous system, inflammatory macrophages have been associated with multiple sclerosis, amyotrophic lateral sclerosis, stroke, spinal cord injury and traumatic brain injury [21–25].

In contrast, murine M2(IL-4) macrophages up-regulate mannose receptor (Mrc1, a.k.a. CD206) and have increased phagocytic and antigen presentation capabilities relative to M1 cells [26]. Murine M2(IL-4) macrophages also strongly up-regulate arginase-1 (Arg-1), shifting arginine metabolism into polyamines including ornithine and urea [13,27]. This is an alternative pathway to the induction of iNOS and is less toxic to microbes and vulnerable post-mitotic host cells (e.g., neurons) [28]. A switch from M1-like to M2-like macrophages is thought to occur during natural resolution of inflammation and, as a result, M2-like macrophages are often described as having anti-inflammatory or reparative functions. However, excessive or uncontrolled M2-like macrophage activity may cause diseases such as fibrosis or asthma [20].

Understanding the mechanisms that control macrophage gene transcription may lead to new tools or therapies that can be used to manipulate divergent macrophage populations *in vivo*. MicroRNAs (miRNAs) are small RNAs, 19 to 24 nucleotides in length, that act as master regulators of gene expression, differentiation and cell function [29,30]. miRNAs inhibit protein translation and/or induce mRNA degradation by binding complementary sequences on the 3’ untranslated region (UTR) of target gene mRNA [31]. Emerging data indicate that miRNAs control large transcriptional networks associated with immune cells and CNS resident microglia [32–38].

Here, we report that miR-155 is critically important for controlling the signature of inflammatory M1(LPS + IFN-γ) macrophages. Indeed, macrophages from miR-155 knockout (KO) mice as well as wild-type (WT) macrophages treated with a miR-155 oligonucleotide inhibitor failed to express M1 macrophage markers, including Nos2, *Tnfa* and *Il1b* in response to stimulation with LPS + IFN-γ. Comparative transcriptional profiling of unstimulated and M1(LPS + IFN-γ) macrophages derived from wild-type (WT) and miR-155 knockout (KO) mice revealed that half (approximately 650 genes) of the signature previously identified in WT M1(LPS + IFN-γ) macrophages [17] was dependent on miR-155. We confirmed that miR-155 is required to suppress validated miR-155 targets *Inpp5d*, *Tspan14*, *Ptprj and Mafb* in M1 macrophages. The loss of these miR-155 targets in inflammatory M1(LPS + IFN-γ) macrophages may mediate miR-155 dependent increases in inflammatory mediators and costimulatory/adhesion molecules. In conclusion, we have identified miR-155 as a pivotal regulator of the M1(LPS + IFN-γ) inflammatory macrophage signature and a potential therapeutic target in inflammatory diseases.

## 2. Materials and Methods

### 2.1. Mice

Wild-type (WT) or miR-155 KO (B6.Cg-*Mir155tm1*.*1Rsky*/J) mice on the C57BL/6J background (Jackson Laboratories) were bred and kept in specific pathogen-free conditions at The Ohio State University Laboratory Animal Resources. All animal experiment procedures were approved under Ohio State University’s IACUC protocol # 2009A0036-R1 and 2013A00000151 to ensure the humane care and use of animals. Euthanasia was performed by cervical dislocation after ketamine/xylazine anesthesia or CO_2_ treatment.

### 2.2. Bone marrow derived macrophages (BMDM)

To generate BMDM, the bone marrow cells from femurs and tibias from mice were harvested and cultured as previously described [25]. Briefly, isolated cells were incubated in Dulbecco’s Modified Eagle Media (DMEM, Mediatech, Herndon, VA) supplemented with 10% heat-inactivated fetal bovine serum (FBS) (Life Technologies, Grand Island, NY)), 1% penicillin/streptomycin, 1% glutamine, and 20% L929 cell supernatant (containing macrophage colony stimulating factor). On day 7 in culture the cells were counted and replated at a density of 0.5–1.0x10^6^ cells/well (24-well plate). No significant differences in CD11b^+^F480^+^ percentage were observed between WT and miR-155 KO BMDMs (WT: 80 ± 2.3, n = 4, KO: 78 ± 1.5, n = 4, t test p = NS; one experiment representative of 3 independent experiments with n≥3 each). Cells were classically or M1-activated (M1(LPS + IFN-γ) condition) with LPS (10 mg/ml, Sigma-Aldrich) + IFN-γ (20ng/mL, E-bioscience, San Diego, CA) or alternatively/M2-activated (M2(IL-4) condition) with IL-4 (20ng/mL, E-bioscience) or received media alone (unstimulated M0 condition) for 24 hours. Cells were harvested at the indicated time-points for RNA isolation, protein isolation or flow cytometry. The 24-hour time-point was chosen as it has been shown by us and others to be sufficient for expression of murine M1 and M2 markers [11,17,39] and fits with the first-line-of-defense function of innate immune cells. Later time-points were not ideal as untreated macrophages kept in culture have been shown to induce M2 marker expression [17].

### 2.3. RNA Isolation

To examine miRNA expression, cells were isolated using the miRVana isolation kit (Life Technologies) according to manufacturer specifications. RNA quality/concentration was quantified using a Nanodrop spectrophotometer (ThermoScientific, Wilmington, DE) and/or Agilent bioanalyzer (Agilent Technologies, Santa Clara, CA). Samples were stored at -80°C until analysis.

### 2.4. miRNA and Real-Time PCR

miRNA expression was determined by Taqman Real-Time PCR using miR-27, miR-29b, miR-155, miR-223, miR-124 and sno-202 primer and probe sets (Life Technologies), according to manufacturer’s instructions. Briefly, after an initial cDNA transcription using specific miRNA primers to generate cDNA using 10 ng RNA as a template, PCR was performed using Taqman universal PCR mix and gene-specific miRNA primers and probe mixture. Reaction mixture was run in an Applied Biosystems 7900 Real-Time PCR machine with denaturation step at 95°C for 10 minutes, followed by 40 cycles of denaturation at 95°C for 15 seconds and primer annealing/extension at 60°C for 60 seconds. miRNA expression was normalized to the small RNA sno202.

mRNA gene expression was determined using SYBR Green or Taqman quantitative Real-Time PCR on cDNA template. cDNA was generated from 500–1000 ng RNA per sample using random hexamer primers (pN6) and Superscript II (Life Technologies), according to manufacturer’s instructions. Product was amplified with 0.5 μM forward and reverse primers of gene of interest and SybrGreen Mastermix (Life Technologies) or with Taqman primers and probe sets and Taqman Mastermix (Life Technologies) on an Applied Biosystems 7900 Real-Time PCR. The primer sequences for SybrGreen primer sets were the following: *Nos2* (F: GGCAGCCTGTGAGACCTTT; R: TTGGAAGTGAAGCGTTTCG), *Il1b* (F: CAGGCTCCGAGATGAACAAC; R: GGTGGAGAGCTTTCAGCTCATAT) and *Tnfa* (F: CTGTGAAGGGAATGGGTGTT; R: GGTCACTGTCCCAGCATCTT). All other genes used Taqman primer and probe sets commercially available from Applied Biosystems. Expression of target genes was normalized to hypoxanthine guanidine phosphoribosyltransferase (*Hprt*) as a loading control. Real-Time PCR data was analyzed using the comparative Ct (ΔΔCT) method or the standard curve method depending on whether the test gene and *Hprt* gene amplification efficiencies were comparable or not [40,41].

### 2.5. Protein analysis

Lysates and media were collected for multiplex protein array analysis. Lysates were collected in RIPA buffer + 0.5% bovine serum albumin. Samples were analyzed using a system that uses microbeads and flow cytometry (Bio-Plex Suspension Array System, Bio-Rad Laboratories Inc., Hercules, CA). Fluorescent-coded beads are conjugated to defined antibodies that recognize the cytokines/chemokines in this quantitative technique. The cell lysates or control media were first incubated for 90 min with all of the microbeads types. After washing, the samples were incubated with biotinylated secondary antibodies also specific for the target cytokines for 30 min. The samples were washed again, incubated with streptavidin-coupled phycoerythrin reporter for 10 min, and then subjected to a final wash. The samples were then diluted in buffer and underwent flow cytometry analysis. At least 100 microbeads were assayed for every sample. The concentrations of each cytokine were calculated based on the inclusion of a standard curve with defined amounts of every analyte.

### 2.6. Flow cytometry

The bone marrow, lymph nodes and spleen of WT and miR-155 KO mice (n = 6-9/mice/group, three independent experiments) were harvested and processed to a single cell suspension. Cells were blocked with anti-mouse FcR antibody (CD16/CD32, BD, Product # 553141) for 15 min at 4°C in FACS buffer (PBS with 2% FBS and 1 mM EDTA) and subsequently surface stained with antibodies for CD11b (clone M1/70 or IgG2b, k isotype, Biolegend), CD11c (Clone N418 or Armenian hamster IgG isotype, BD), Ly6C (clone HK1.4 or rIgG2a, k isotype, Biolegend) and Ly6G (clone 1A8 or rIgG2a, k isotype, Biolegend), F480 (clone BM8 or rIgG2a, k isotype, Biolegend) for 15 min at 4°C. Cells were washed three times with FACS buffer and run through a BD FACSCanto Flow Cytometer (BD, NJ). Data was analyzed with FlowJo (Treestar, OR).

### 2.7. miRNA mimic and inhibitor transfection

Mouse bone marrow-derived macrophages were transfected overnight with 200 nM of double-stranded Power miRNA inhibitors (Exiqon Inc, Woburn, MA) or 7 nM single stranded precursor miRNA mimics (ThermoFisher) using the Transit-TKO Reagent (Mirus Bio LLC, Madison, WI). Power Inhibitor sequence were GTGTAACACGTCTATACGCCCA for nonsense control (NS) (Exiqon 199020–00) and GTGTAACACGTCTATACGCCCA for miR-155 (Exiqon 428232–00). miRNA mimic sequences/catalog number were AM17111 for miR control and UUAAUGCUAAUUGUGAUAGGGGU/AM17100 for miR-155. Cells were transfected for approximately 18 hours before transfection reagents were removed and macrophages received exogenous cytokines to stimulate differentiation. Macrophages received either LPS (10 ng/mL) + IFN-γ (20 ng/mL) to stimulate the M1(LPS+ IFN-γ) condition, IL-4 (20ng/mL) to stimulate the M2(IL-4) condition, or media alone for the unstimulated (M0) condition. Cells were subsequently lysed for RNA 24 hours post-stimulation.

### 2.8. Microarray

Total RNA was prepared from bone marrow-derived macrophages of 3 WT and 3 miR-155 KO mice treated in M0 or M1 conditions (as defined in M&M section 2.2) for 24 hours using the miRVana isolation kit (Ambion). RNA quality was analyzed by the RNA 6000 Nano Chip (Agilent, and only samples with an RNA Integrity Number (RIN) >7 were used for further processing. One KO sample had insufficient RNA quality for microarray and was therefore removed from analysis. RNA was processed and hybridized to the Affymetrix Mouse 430 2.0 chips at the Ohio State University Comprehensive Cancer Center (OSUCC) Microarray facility. Raw data were normalized with the RMA algorithm implemented in the ‘‘Expression File Creator” module from the GenePattern software package [42]. Data were visualized with the Multiplot modules from GenePattern. Array data are deposited at the Gene Expression Ommibus (GEO) NCBI database with accession numbers GSE69607 (WT) and GSE77452 (KO).

### 2.9. Ingenuity Pathway Analysis

A gene list was compiled from the Affymetrix array results for ingenuity pathway analysis (IPA) using the genes that had a ≥2 fold change (FC) difference between WT M1 vs. WT M0 but a <2FC between KO M1 vs. KO M0 macrophages [17] plus genes that had a ≥2FC between WT M1 and KO M1 macrophages. A Core Analysis was run on this data set to determine the pathways most affected by the loss of miR-155. Additionally, the miR Target Filter was used to identify potential direct miR-155 targets from the full list of microarray probes, identified as any genes that were down-regulated in the WT M1 vs. WT M0 were considered potential direct targets of miR-155.

### 2.10. Statistical analysis

Statistical significance was determined using unpaired t-test (two-tail, equal SD) or analysis of variance (ANOVA) followed by Tukey post-hoc test. For microarray analysis, p values were Benjamin-Hochberg adjusted for multiple comparisons. Statistical significance was determined to be p<0.05. Analysis was completed using GraphPad Prism or GenePattern.

## 3. Results

### 3.1. miR-155 is selectively up-regulated in classically activated M1(LPS + IFN-γ) macrophages

**Fig 1:**
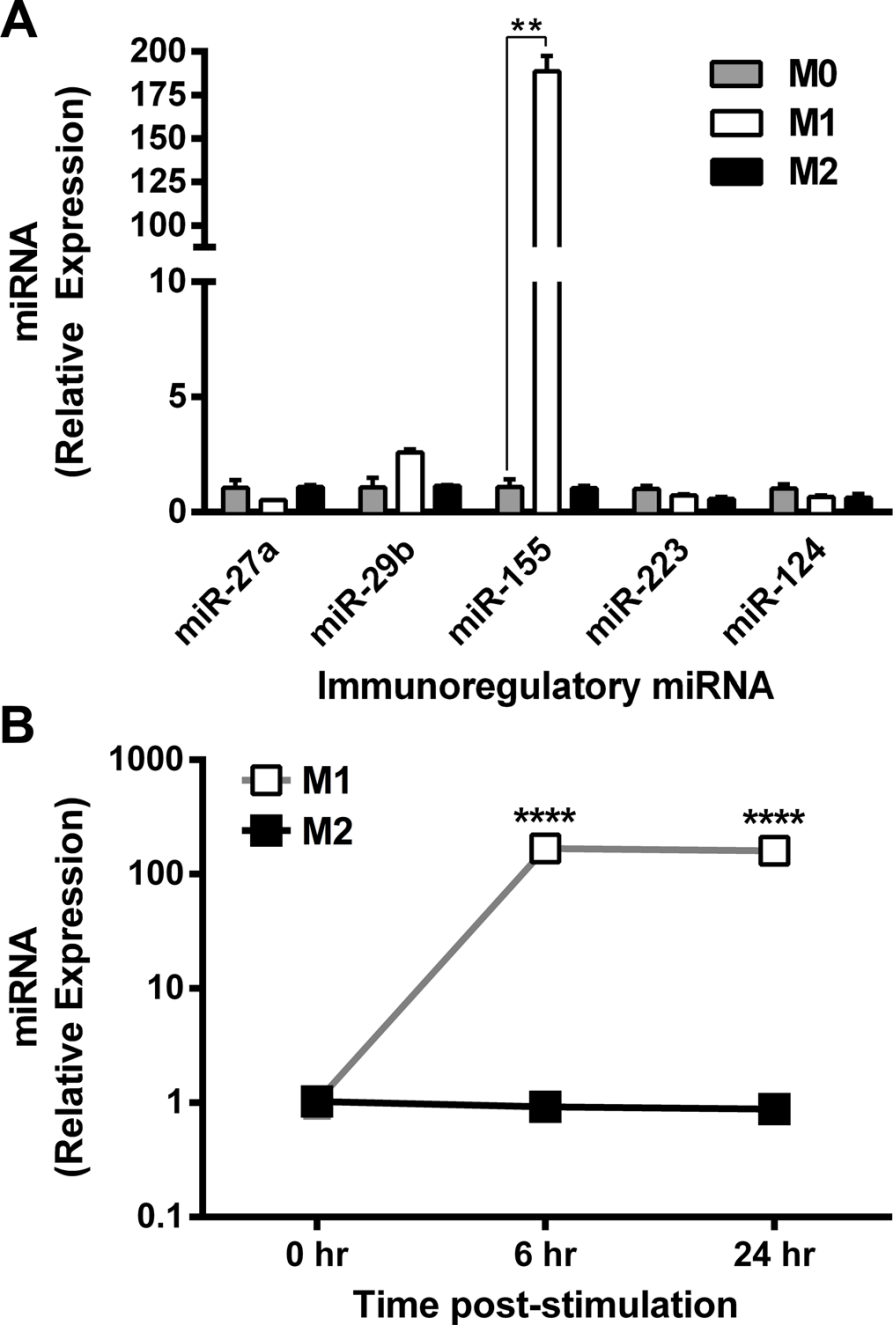
miR-155 is associated with the classically activated macrophage phenotype. Expression of miRNAs was determined by Taqman Real-Time PCR and expressed as mean relative expression (+ SEM) in **(A)** macrophages stimulated *in vitro* for 24 hours in M0, M1, and M2 (n = 3) conditions; expression relative to M0 condition; Post-hoc ANOVA, **p<0.005. **(B)** M1 and M2 macrophages activated *in vitro* over a 48 hour period; expression relative to 0 hour pre-stimulation time-point. Unpaired t-test, ***p<0.001. Results reproduced in two independent experiments.

Specific miRNA expression signatures have been associated with discrete cellular lineages or phenotypes [32,36]. We analyzed expression of miR-27b, miR-29b, miR-155, miR-124 and miR-223 in bone marrow-derived mouse macrophages differentiated in M0 (unstimulated), M1(LPS + IFN-γ) and M2(IL-4) conditions. These miRNAs were analyzed because they have been linked to inflammatory responses in which macrophages comprise the primary subtype of responding leukocytes [32,35–37,43]. Of these miRNAs, miR-155 was the most highly up-regulated in response to M1(LPS + IFN-γ) stimulation conditions (fold change (FC) ± standard deviation (SD) = 182 ± 13, ANOVA followed by post-hoc Bonferroni test p< 0.005). In contrast, miR-155 was not up-regulated in M2(IL-4) macrophages (1.0 ± 0.153) (**Fig 1A**).

To reveal the temporal pattern of miR-155 expression during macrophage activation, we quantified the relative expression of miR-155 at 0, 6 and 24 hours post-stimulation (**Fig 1B**). Expression of miR-155 in M1(LPS + IFN-γ) macrophages increased significantly (t-test, p<0.0001) and reached its maximum expression by 6 hours post-stimulation. In contrast, M2(IL-4) macrophages did not up-regulate miR-155 at any of these time points. Collectively, these data indicate that induction of miR-155 is specifically associated with differentiation of classically activated M1(LPS + IFN-γ) macrophages.

### 3.2. Genetic miR-155 deficiency abrogates expression of classically activated M1 macrophage markers

**Fig 2:**
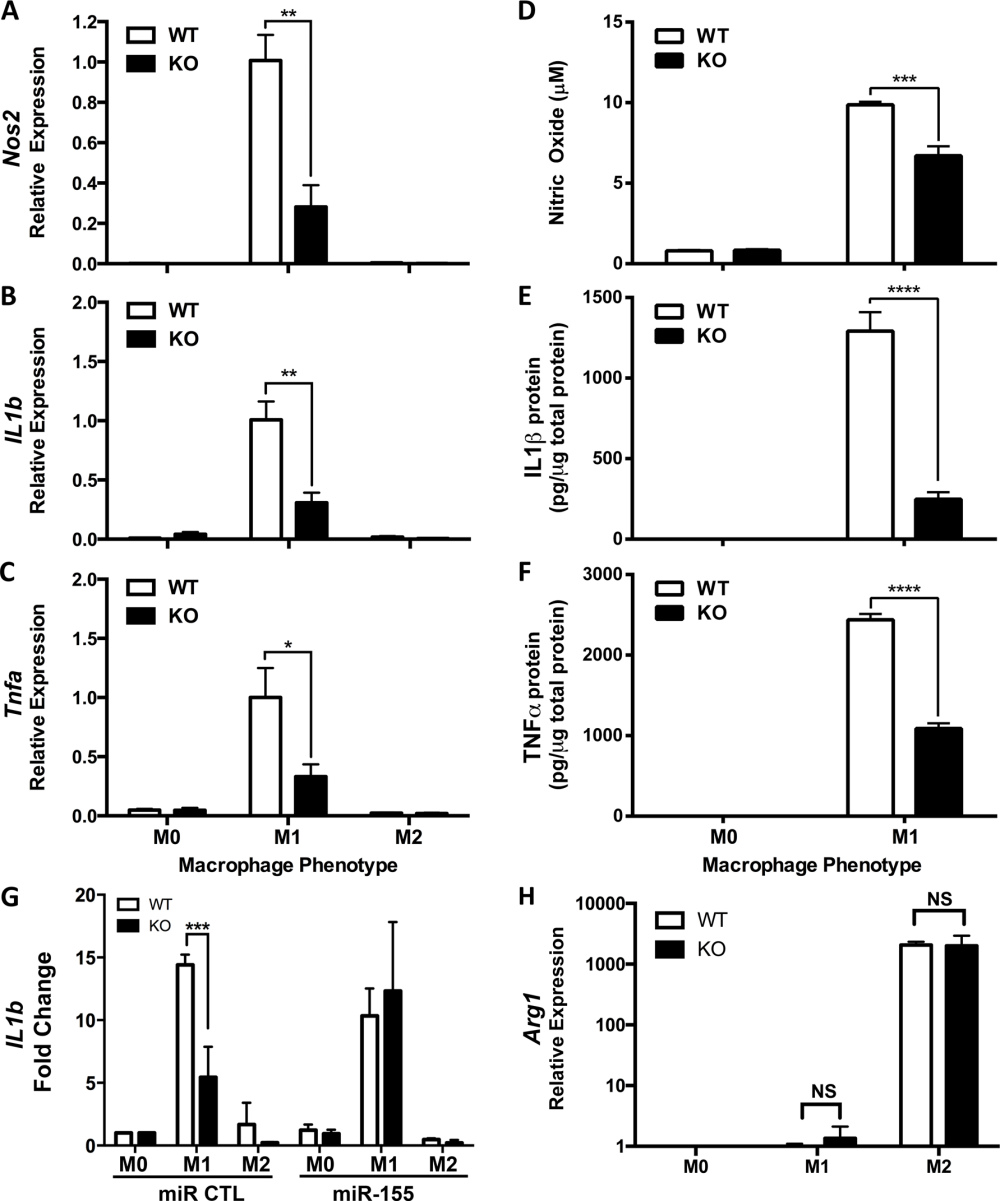
Reduced classically activated M1 marker expression in miR-155 knock-out (KO) macrophages. **(A)** Inducible nitric oxide synthase (*Nos2*), **(B)** *IL1b*, (**C**) Tumor Necrosis Factor-α (*Tnfa*) **(H**) and Arginase-1 (*Arg1*) expression was determined by Real-Time PCR in wild-type (WT, n = 8–11) and miR-155 knockout (KO n = 8–12) bone marrow-derived macrophages *in vitro* activated in M1 or M2 conditions for 24 hours in three independent experiments. Gene expression is expressed as a percentage +/- SEM of the WT M1 condition. Unpaired t-test, *p<0.05, **p<0.005. Relative concentration of **(D)** nitric oxide (NO), **(E)** IL-1β protein and **(F)** TNF-α protein was determined using Griess assay (for NO) or Bio-Plex Suspension Array (For IL-1β and TNF-α) in cell lysates from WT (n = 5) and miR-155 KO (n = 5) bone marrow-derived macrophages *in vitro* activated in M1 or M2 conditions for 24 hours. Individual protein concentrations expressed as fraction of total protein concentration in either M0 or M1 condition. (**G**) *IL1b* expression was determined by Real-Time PCR in WT and KO bone marrow-derived macrophages transfected with a scrambled miR control (n = 5) or a miR-155 oligonucleotide mimic (n = 5) and activated in M0 (untreated), M1 (LPS+IFN-γ) or M2(IL-4) conditions for 24 hours. Gene expression is expressed as a percentage +/- SEM of the scrambled M1 condition. Unpaired t-test, ***p<0.0005, ****p<0.00005. (A-G) Data from 2–3 independent experiments.

Genetic loss-of-function experiments were used to determine whether markers of inflammatory macrophage phenotype are dependent on miR-155 by using the M1(LPS + IFN-γ) M1 macrophage model [8–12]. BMDM isolated from WT (n = 5) or miR-155 KO (n = 5) mice were kept unstimulated (M0) or stimulated in M1(LPS + IFN-γ) or M2(IL-4) conditions for 24 hours. RT-PCR was used to analyze expression of canonical M1 macrophage markers (*Inos*, *Il1b* and *Tnfa*) [9,10] and the M2-associated gene, *Arg1* (**Fig 2**). As expected, *Nos2*, *Il1b* and *Tnfa* were expressed at higher levels in M1(LPS + IFN-γ) vs. M2(IL-4) WT macrophages and *Arg1* was more highly expressed in M2(IL-4) vs. M1(LPS + IFN-γ) macrophages. miR-155 KO macrophages showed striking reductions in expression of the three M1 genes and the proteins/effector molecules they encode (reduced up to 72%) (**Fig 2A–2F**). Restoring miR-155 in miR-155 KO macrophages recovered inflammatory cytokine production (**Fig 2G**). In contrast, expression of the M2 gene, *Arg1*, was not significantly different between WT and KO M2 (IL-4) macrophages (**Fig 2H**, t-test, not significant).

To ensure that impaired gene expression in miR-155-deficient macrophages was not caused by a defect in immune system development in genetically modified mice, we quantified the relative proportion of monocytes, macrophages, dendritic cells and polymorphonuclear cells in different lymphoid tissues of WT and miR-155 KO mice. No significant differences in any myeloid cell population were found in the bone marrow, spleen or lymph nodes (**S1 Fig**). Similarly, we found no differences in the percentages of CD11b^+^F480^+^ percentage obtained for WT and miR-155 KO BMDMs (data not shown).

**Fig 3:**
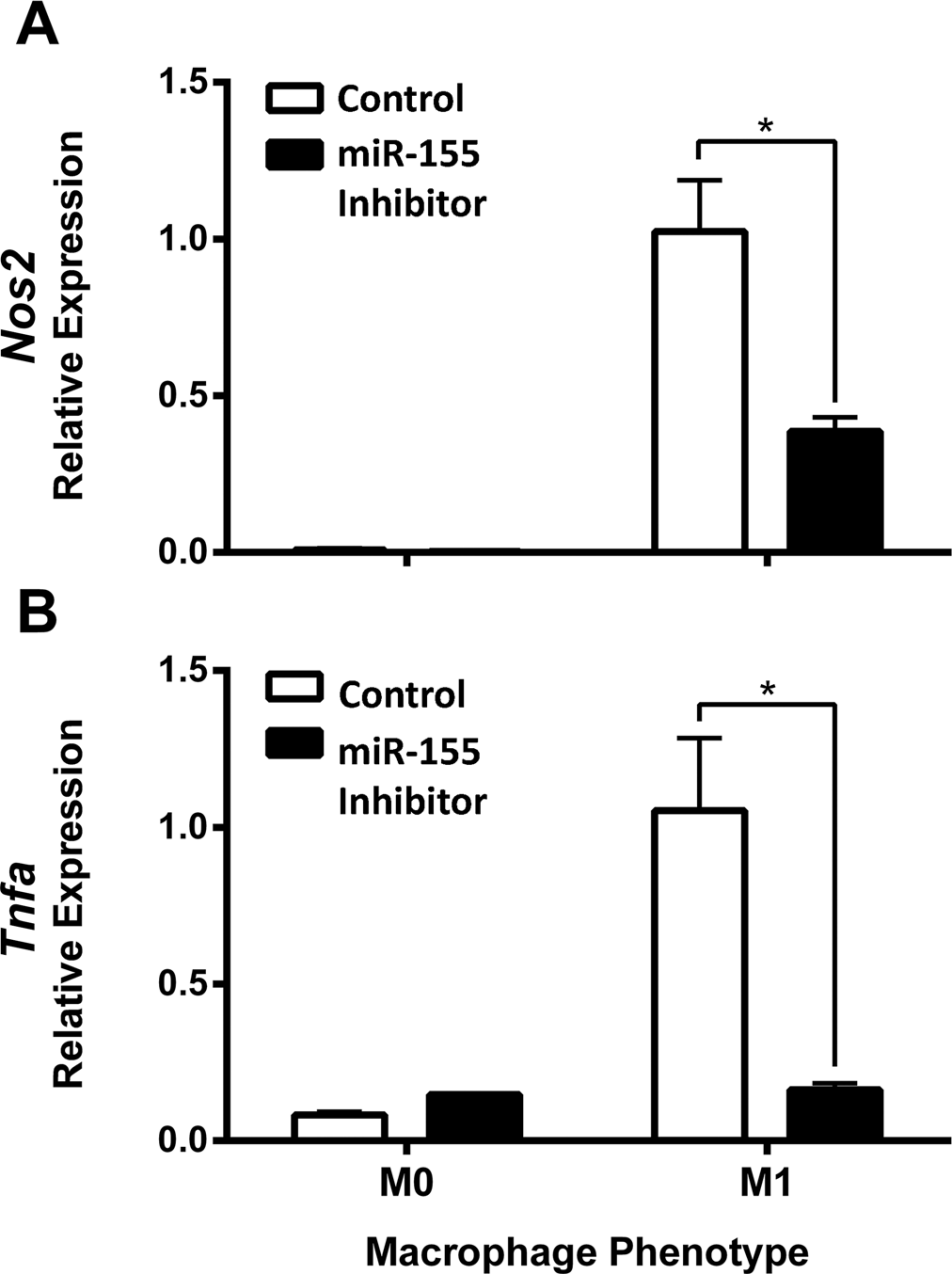
miR-155 inhibitor reduces M1 marker expression. (**A**) Inducible nitric oxide synthase (*Nos2*) and (**B**) Tumor Necrosis Factor (*Tnfa*) expression was determined by Real-Time PCR in wild-type bone marrow-derived macrophages *in vitro* activated in M0 or M1 conditions for 24 hours and transfected with a scrambled (n = 3) or a miR-155 oligonucleotide inhibitor (n = 3–4). Gene expression is expressed as a percentage +/- SEM of the scrambled M1 condition. Unpaired t-test, *p<0.05. Data reproduced in two independent experiments.

Next, an oligonucleotide-based inhibitor was used to block miR-155 in WT macrophages during induction of the M1(LPS + IFN-γ) phenotype. As a control, macrophages were transfected with a scrambled oligonucleotide inhibitor that lacks specificity for miR-155. 24 hours post-activation, expression of *Nos2* and *Tnfa* mRNA was analyzed using RT-PCR. The miR-155 inhibitor suppressed *Inos* and *Tnfa* expression by 75% and 85%, respectively, in M1(LPS + IFN-γ) macrophages as compared to macrophages transfected with the scrambled inhibitor (**Fig 3**).

### 3.3. miR-155 is necessary for the full expression of the M1(LPS + IFN-γ) macrophage signature

**Fig 4:**
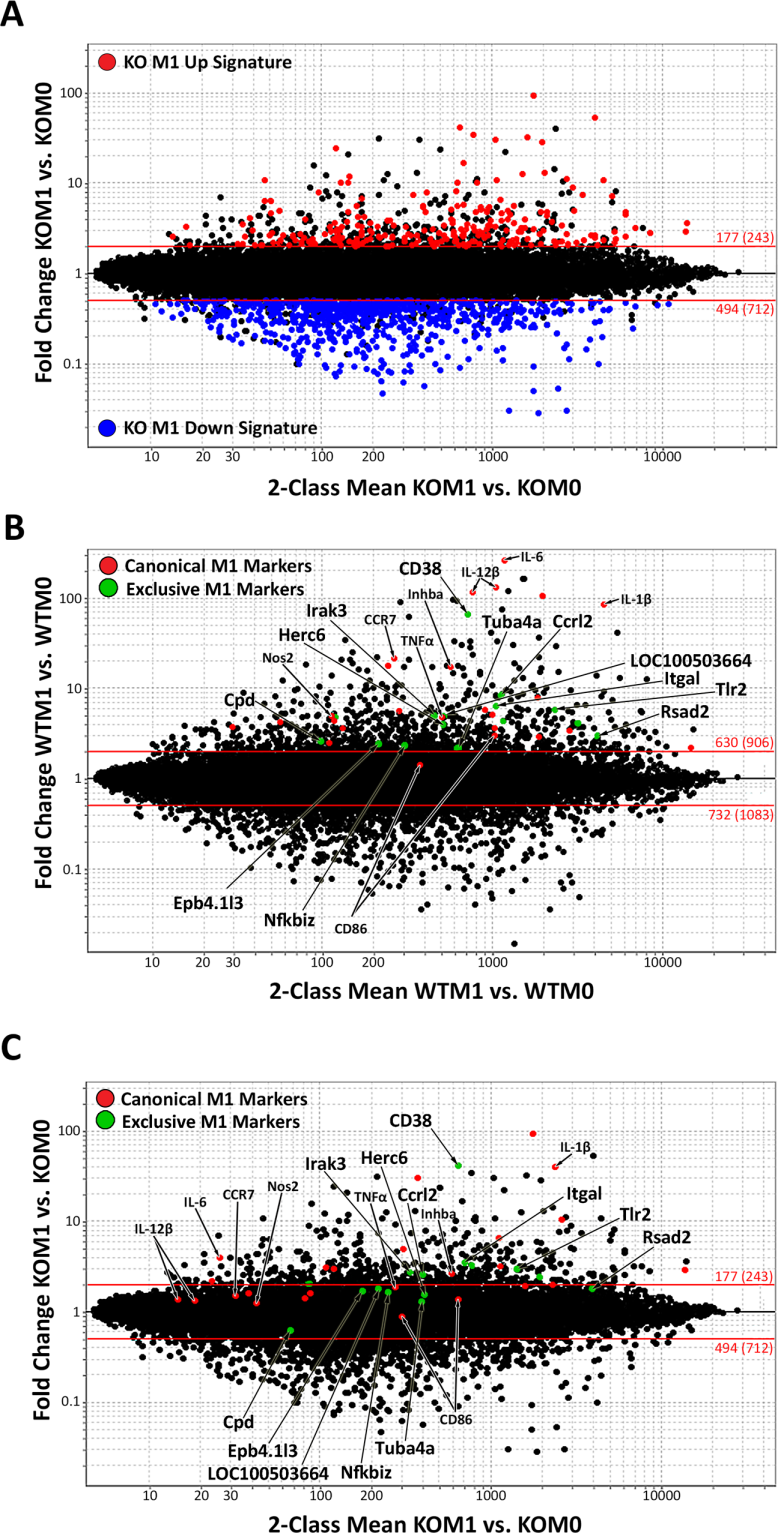
Classically activated macrophage signature in wild-type and miR-155 knockout macrophages. Fold Change (FC) vs. Mean Expression Value (MEV) plot of microarray data highlighting 2 FC or higher up-regulated genes (red, p≤0.05) or down-regulated genes (blue, p≤0.05) in **(A)** knockout **(**KO) M1 to M0 macrophages comparison (n = 3). (**B, C**) FC vs. MEV plot with previously described classical M1 markers highlighted (in red) and the top 15 M1-exclusive genes (identified in [17]) most decreased in KO M1 macrophages (in green) in the WT M1 vs. M0 comparison (**B**) and the KO M1 vs. M0 comparison (**C**). Red lines represent a +/- 2FC cut-off.

To determine the global effect of miR-155 on regulating gene expression in M1 macrophages, we performed gene expression profiling on miR-155 KO macrophages. Affymetrix M430 2.0 arrays were hybridized with complementary DNA (cDNA) isolated from KO macrophages cultured in M0 or M1(LPS + IFN-γ) conditions. **Fig 4A** shows that, when compared to the baseline KO M0 condition, 177 genes (≥2FC, p<0.05, in red, S1 **Table**) were up-regulated and 494 genes (≤0.5FC, p<0.05, in blue, S2 **Table**) were down-regulated in M1(LPS + IFN-γ) KO macrophages. When compared to WT macrophages activated under identical conditions [17], ~700 fewer genes (or ~50%) changed in miR-155 KO macrophages.

To determine the impact of miR-155 on the canonical M1 phenotype, we compared the induction of canonical M1(LPS + IFN-γ) genes (full list and FC in S3 **Table**) between WT (**Fig 4B, in red**) and miR-155 KO macrophages (**Fig 4C, in red)**. The majority of M1 markers (22 out of 25 gene probes) decreased their expression in KO vs. WT M1(LPS + IFN-γ) macrophages. In most cases, these genes were found under the 2FC line (**Fig 4C**), indicating that their induction is highly dependent on miR-155. We also evaluated the effect of miR-155 loss on 15 genes that we recently found to be exclusively upregulated in M1(LPS + IFN-γ) macrophages [17]. These genes are highlighted in green in **Fig 4B and 4C**. Since the markers examined correspond to only a small portion of the M1(LPS + IFN-γ) signature, it is likely that miR-155 has more wide-ranging effects on the inflammatory M1 signature.

**Fig 5:**
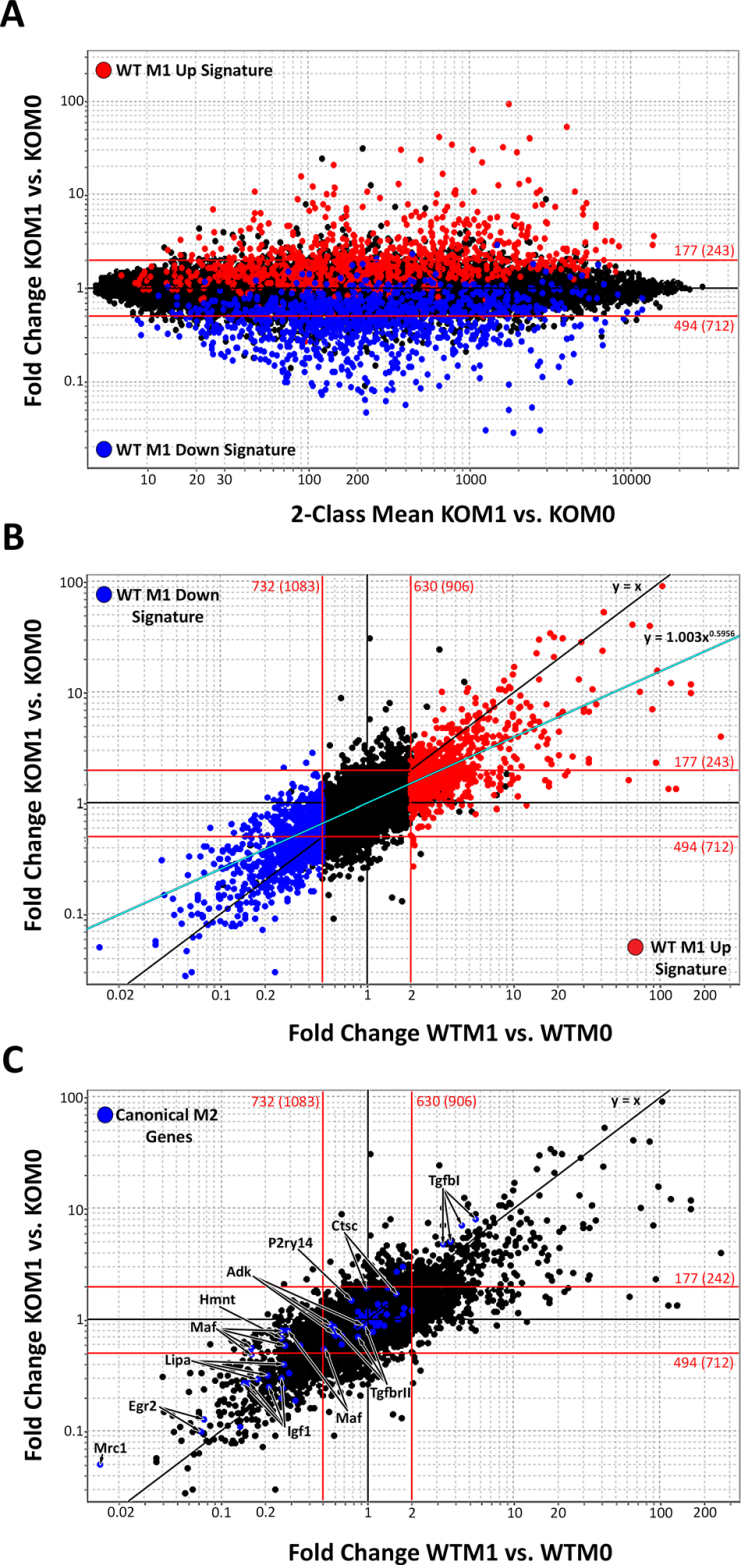
miR-155 is required for expression of the full classically activated macrophage signature. (**A**) Fold Change (FC) vs. Mean Expression Value (MEV) plot of knockout (KO) M1 vs. M0 microarray data, with highlighted classical wild-type (WT) M1 signature genes as defined in this study. Genes more than 2FC up-regulated in WT M1 macrophages (red genes in **Fig 4A**) are shown in red and genes more than 2FC down-regulated in WT M1 macrophages (blue genes in **Fig 4A**) are shown in blue. (**B**) WT M1/M0 FC vs. KO M1/M0 FC plot highlighting the WT M1 Up signature (in red) and WT M1 Down signature (in blue). The black line indicates the *x = y* trendline expected if all gene probes were similarly up- or down-regulated exactly the same in KO M1 and WT M1 macrophages (FC KO M1 vs KO M0 = FC WT M1 vs WT M0). The blue line indicates the power regression trendline that genes more closely adhered to, represented by *y = 1*.*003x*^*0*.*5956*^, R^2^ = 0.50052. (**C**) WT M1/M0 FC vs. KO M1/M0 FC plot highlighting classical M2 genes. Red lines represent a +/- 2FC cut-off. More than a single appearance of a gene symbol indicates different probes hybridizing the same gene transcript were present.

To reveal the scope of the M1(LPS + IFN-γ) macrophage signature that is completely dependent on miR-155 (no longer 2FC up- or down-regulated in KO macrophages), the previously defined WT M1(LPS + IFN-γ) signature [17] (M1 Up Signature shown in red and M1 Down Signature shown in blue) was highlighted on the KO M1 vs. M0 plot (**Fig 5A**). Most of the WT M1(LPS + IFN-γ) signature (about 72% of the WT M1 Up gene signature and 33% of the WT M1 Down signature) fell within the 2FC lines. Overall, miR-155 deficiency resulted in loss of 51% of the M1 signature.

Utilizing standard 2FC threshold values (red lines in **Fig 5A**) is helpful to identify WT M1(LPS + IFN-γ) signature genes that fully require miR-155 for expression. However, we suspected miR-155 also had subtler, but no less important, quantitative effects on the inflammatory M1 signature. For example, we observed a ~260 FC increase in IL-6 expression in WT M1 condition as opposed to only a ~4 FC increase in KO M1 condition. This important change in IL-6 would have been overlooked in the previous analysis because its expression remains above the 2FC line in both the WT and KO M1 signatures. To reveal these additional effects of miR-155 deletion, we used a FC vs. FC plot. FC vs. FC plots compare the magnitude of gene expression changes brought about by M1(LPS + IFN-γ) activation in WT macrophages or KO macrophages. Genes that are induced/repressed to the same extent in WT and KO macrophages fall on the *y = x* line, while genes that are induced/repressed to a different extent will deviate from this line. When the data were plotted on a FC vs. FC plot (**Fig 5B**), we observed that the gene population data deviated from the *y = x* trend line, adjusting instead to the *y = 1*.*003x*^*0*.*5956*^ trend line (represented in teal blue in **Fig 5B**). This shift in the gene expression trend line shows that genes that are up- or down-regulated in WT M1 conditions are changed to a lesser extent in miR-155 KO macrophages. This indicates that, besides the large ≥2FC effects previously identified in **Fig 4**, miR-155 has a widespread dampening effect on the magnitude of gene expression changes caused by macrophage stimulation in M1 conditions.

### 3.4. Increased alternatively activated macrophage gene expression in classically activated miR-155 deficient macrophages

We next determined whether the KO M1(LPS + IFN-γ) macrophage phenotype resembled the opposing M2 macrophage phenotype, since macrophage phenotype is thought to be dependent on gene expression [6,7]. To determine if miR-155 loss promotes an M2 macrophage phenotype under inflammatory conditions, we highlighted a number of previously described M2 macrophage markers [14] (in blue, listed in S4 **Table**) on the FC vs. FC plot (**Fig 5C**). As expected, most genes were down-regulated in the WT M1 vs. WT M0 FC comparison. A subset of these genes was more highly expressed (above the *y = x* line) in KO than in WT M1(LPS + IFN-γ) macrophages (FC values listed in S4 **Table**). These genes included mannose receptor 1 (Mrc1), early growth response 2 (Egr2), cathepsin c (Ctsc), purinergic receptor P2Y (P2ry14), avian musculoaponeurotic fibrosarcoma (v-maf) AS42 oncogene homolog (C-Maf), histamine N-methyl transferase (Hnmt), adenosine kinase (Adk), insulin-like growth factor (Igf1), TGF-β receptor 2 (Tgfbr2) and lysosomal acid lipase A (Lipa). This indicates that loss of miR-155 gene regulation prevents the full down-regulation of M2(IL-4) genes that normally occurs under the influence of strong inflammatory activation signals including IFN-γ and LPS.

### 3.5. The miR-155 dependent M1(LPS + IFN-γ) signature is enriched in inflammatory signaling pathways

**Fig 6:**
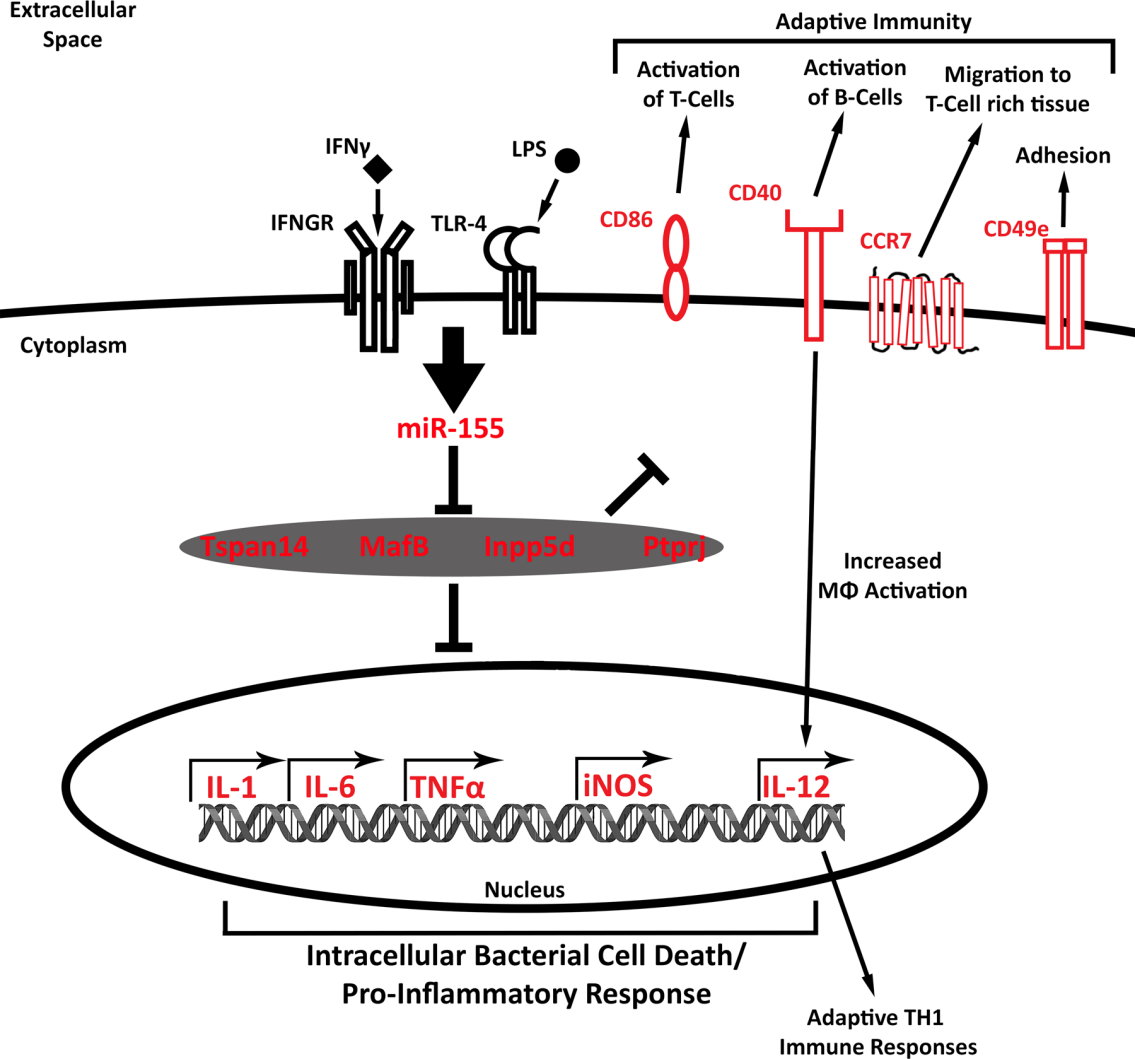
miR-155 dependent M1 signature transcriptional networks. (A). Model of molecules involved in miR-155 dependent M1 activated transcriptional networks, identified by Ingenuity Pathway Analysis. miR-155-dependent M1 genes up-regulated more than 2 Fold Change (FC) in wild-type (WT) M1vs. WT M0 macrophages that were up-regulated to a lesser extent in knockout (KO) M1 vs. KO M0 macrophages are highlighted in red.

The mRNA profiling data provided an opportunity to identify basic M1 macrophage “functions” that are dependent on miR-155 expression. Ingenuity Pathway Analysis (IPA) was applied to the 2FC Up and Down signature M1(LPS + IFN-γ) signature genes that were no longer induced or repressed in KO M1(LPS + IFN-γ) macrophages, revealing that the genes promoted by miR-155 (red molecules designated in **Fig 6**) encode proteins critical for pathways involved in bactericidal functions, inflammatory responses and costimulation and enhancement of B and Th1 T lymphocyte responses. Several genes involved in TLR and IFN-γ receptor (IFN-γR) signaling also were regulated by miR-155. Among these were Janus kinase 2 (Jak2), involved in IFN-γR signaling, and interleukin-1 receptor-associated kinase 1 (Irak2 and 3) and V-Akt Murine Thymoma Viral Oncogene Homolog 1 (Akt), involved in TLR signaling. Additionally, expression of downstream effector molecules associated with inflammatory responses and bacterial killing, such as IL-1β, IL-6, TNF-α, NO and IL-12, were miR-155 dependent. Similarly, expression of several membrane molecules involved in macrophage-mediated stimulation of adaptive inflammatory responses (e.g., CD40, CD86, CD49e and Ccr7) were increased in WT M1(LPS + IFN-γ) but were not induced in KO M1(LPS + IFN-γ) macrophages.

### 3.6. Discovery of miR-155 target mRNAs repressed in M1(LPS + IFN-γ) macrophages

**Fig 7:**
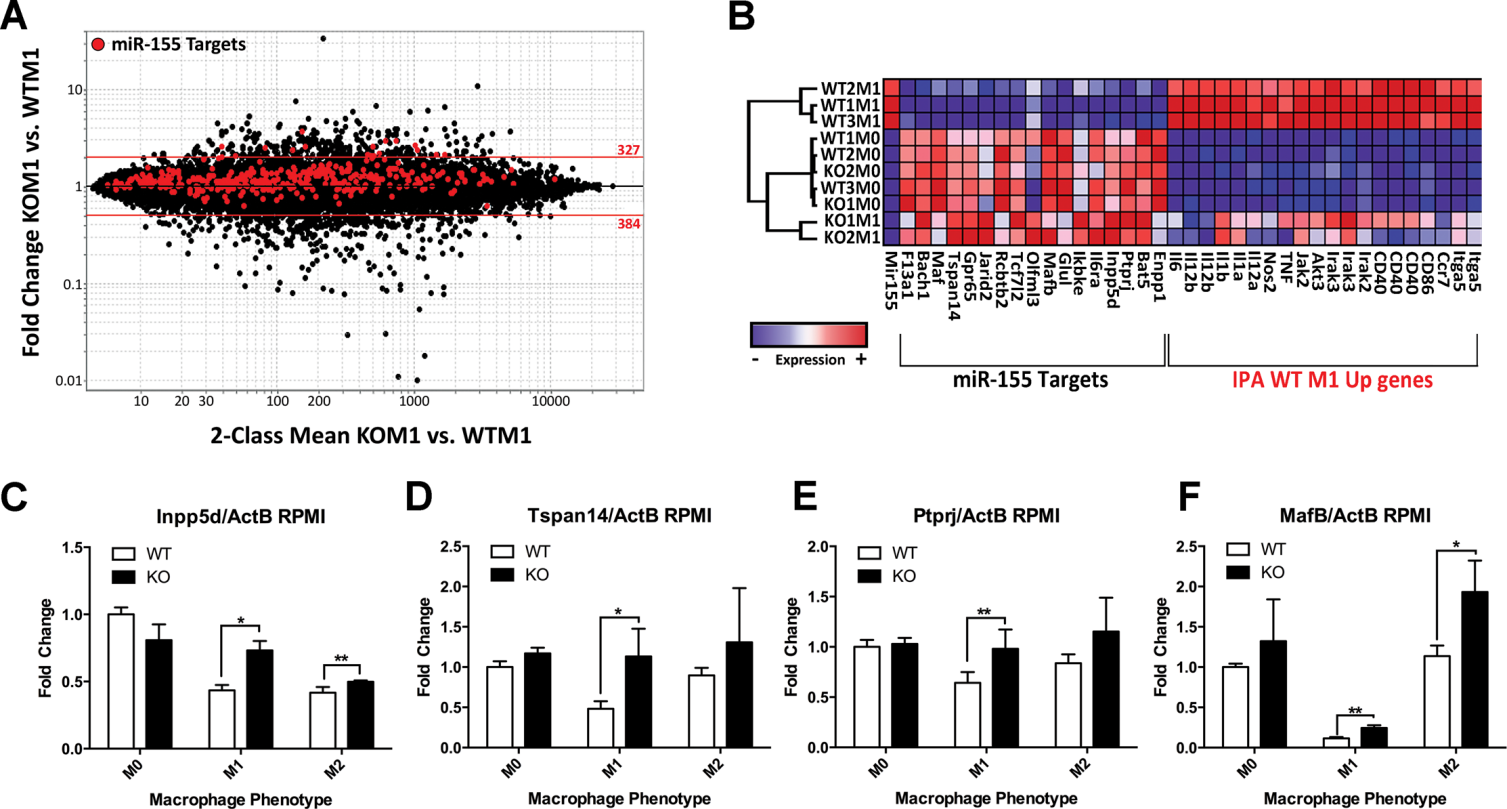
Identification of candidate miR-155 targets associated with M1 phenotype. (**A**) Fold Change (FC) vs. Mean Expression Value (MEV) plot of knockout (KO) M1 vs. wild-type (WT) M1 microarray data with miR-155 targets (high or moderate predicted targets and experimentally observed targets) that were down-regulated ≥2FC in WT M1 vs. WT M0 highlighted in red. 18 target genes were up-regulated more than 2 FC in KO M1 vs. KO M0. (**B**) Hierarchical clustering analysis of WT M0, WT M1, KO M0 and KO M1 samples based on expression of miR-155, IPA identified miR-155 dependent M1 Up genes from **Fig 6** and top 18 miR-155 targets identified in (A). (**C**) *Inpp5d*, (**D**) *Mafb*, **(E**) *Tspan14*, and (**F**) *Bat5* expressions were determined by Real-Time PCR in wild-type (WT, n = **3**) and miR-155 knockout (KO n = **3**) bone marrow-derived macrophages *in vitro* activated in M1 or M2 conditions for 24 hours. Data shown is representative of 2–3 independent experiments. Gene expression is expressed as a percentage +/- SEM of the WT M0 condition. Unpaired t-test, *p<0.05, **p<0.005. (**F**)

miRNAs generally regulate gene expression via binding to the 3’UTR of specific gene transcripts and repressing their expression. To identify miR-155 targets that promoted the inflammatory M1(LPS + IFN-γ) phenotype when repressed, we searched for experimentally observed or high/moderate probability predicted miR-155 targets using the miR Target Filter function in IPA. To filter the list based on potential importance in inducing the M1(LPS + IFN-γ) phenotype, we first focused on the 370 genes (S5 **Table**) down-regulated in WT M1 vs. WT M0 macrophages. When expression of these 370 candidate miR-155 target genes was compared between KO M1(LPS + IFN-γ) and KO M0 samples, we observed that ~80% of these genes were up-regulated (i.e., re-induced) to some extent in the absence of miR-155 (highlighted in red in **Fig 7A**) (FC>1; KO M1 vs. WT M1 condition) (**Fig 7A**) and 18 genes were more than 2FC up-regulated (**Table 1**). 16 of these genes were significantly inversely correlated (Pearson correlation, p<0.05) with miR-155 (**Table 1**).

**Table 1:** miR-155 targets up-regulated more than 2 fold-change in KOM1(LPS + IFN-γ) vs. WTM1(LPS + IFN-γ) macrophages.

Gene		miR-155 Correlation		WTM1 vs WTM0		KOM1 vs WTM1		KOM1 vs KOM0	
Symbol	Description	R	p	FC	p	FC	p	FC	p
**Bat5**	HLA-B associated transcript 5	-0.96	0.0001	0.51	0.0027	2.07	0.0015	1.10	0.261
**Mafb**	v-maf musculoaponeurotic fibrosarcoma oncogene family, protein B (avian)	-0.95	0.0001	0.37	0.0001	2.37	0.0385	0.91	0.599
**Bach1**	BTB and CNC homology 1	-0.94	0.0001	0.40	0.0003	2.95	0.0082	1.09	0.559
**Maf**	avian musculoaponeurotic fibrosarcoma (v-maf) AS42 oncogene homolog	-0.93	0.0001	0.16	0.0024	2.92	0.0221	0.49	0.023
**Ptprj**	protein tyrosine phosphatase, receptor type J	-0.90	0.0003	0.50	0.0082	2.09	0.0103	1.10	0.433
**Il6ra**	interleukin 6 receptor, alpha	-0.90	0.0004	0.49	0.0013	2.14	0.0162	1.21	0.441
**Gpr65**	G-protein coupled receptor 65	-0.87	0.0008	0.52	0.0103	2.58	0.0051	1.35	0.055
**Tspan14**	tetraspanin 14	-0.85	0.0014	0.54	0.0001	2.65	0.0008	1.36	0.027
**Inpp5d**	inositol polyphosphate-5-phosphatase D	-0.84	0.0022	0.64	0.0012	2.11	0.0002	1.34	0.005
**Tcf7l2**	transcription factor 7-like 2, T-cell specific, HMG-box	-0.83	0.0029	0.49	0.0034	2.52	0.0500	1.41	0.267
**Prkar1b**	protein kinase, cAMP dependent regulatory, type I beta	-0.82	0.0034	0.61	0.0079	2.05	0.0311	1.29	0.283
**Rcbtb2**	regulator of chromosome condensation (RCC1) and BTB (POZ) domain containing protein 2	-0.79	0.0058	0.20	0.0150	2.56	0.0099	0.45	0.106
**Enpp1**	ectonucleotide pyrophosphatase/phosphodiesterase 1	-0.77	0.0088	0.22	0.0002	2.03	0.0100	0.42	0.032
**Glul**	glutamate-ammonia ligase (glutamine synthetase)	-0.65	0.0393	0.26	0.0021	2.20	0.0264	0.56	0.036
**F13a1**	coagulation factor XIII, A1 subunit	-0.65	0.0393	0.19	0.0333	3.71	0.0761	0.50	0.406
**Jarid2**	jumonji, AT rich interactive domain 2	-0.64	0.0434	0.64	0.0015	2.56	0.0000	1.87	0.002
**Ikbke**	inhibitor of kappaB kinase epsilon	-0.54	0.1038	0.80	0.0780	2.15	0.0237	1.73	0.073
**Olfml3**	olfactomedin-like 3	-0.26	0.4596	0.96	0.8588	2.39	0.0350	3.11	0.051

R: Pearson correlation R value; FC: Fold Change; p: p value

To visualize the relationship between miR-155, the miR-155 repressed genes identified in **Table 1** and the M1(LPS + IFN-γ) up-regulated genes identified through IPA analysis, a hierarchical clustering analysis was performed on these genes. This highlighted the strong inverse relationship between miR-155 and its repressed genes, as well as an inverse relationship between miR-155 repressed genes and IPA-selected M1(LPS + IFN-γ) Up genes (**Fig 7B**). Interestingly, KO M1(LPS + IFN-γ) macrophages clustered more closely with WT M0 or KO M0 macrophages than with WT M1(LPS + IFN-γ) macrophages through this analysis, suggesting a link between the pattern of expression of this group of genes and the switch from M0 to M1(LPS + IFN-γ) inflammatory phenotype that is mediated by miR-155.

Real-Time PCR on independent datasets confirmed that miR-155 contributed to suppression of its validated targets *Inpp5d*, *Tspan14*, *Ptprj* and *Mafb* (**Fig 7C–7F**, respectively) in M1(LPS + IFN-γ) macrophages. miR-155 deficiency restored *Inpp5d*, *Tspan14* and *Ptprj* expression to WTM0 levels indicating that miR-155 is required for their suppression in M1(LPS + IFN-γ) macrophages. Overall, these data identify various candidate genes that may mediate inflammatory M1(LPS + IFN-γ) phenotype when repressed by miR-155.

## 4. Discussion

Inflammatory macrophages can cause inflammatory disease and tissue damage. Therefore, understanding the molecular programs that control inflammatory M1 phenotype may provide novel targets for therapeutic intervention. Here, we show that rapid and robust M1(LPS+ IFN-γ)-selective up-regulation of miR-155 promotes *Nos2*, *Tnfa* and *Il1b* inflammatory gene expression, as well as their protein products. miR-155 had a strong influence on gene expression, controlling half of the 2FC M1(LPS + IFN-γ) signature, as well as milder widespread effects, modulating smaller gene expression shifts in the remainder of the M1(LPS + IFN-γ) signature. Among the top miR-155 inversely correlated genes that may mediate these effects, we identified and validated miR-155 targets *Inpp5d*, *Tspan14*, *Ptprj* and *MafB*.

miRNA play a critical role in shaping cellular phenotype [44]. Within the immune system, miRNA can modulate the development of opposing Th1 versus Th2 T helper cell phenotypes that mediate autoimmune and allergic disease [32,43]. However, the role of miRNA in controlling macrophage differentiation and effector functions is just beginning to be elucidated. Among 5 miRNA important in inflammatory or LPS responses [32,35–37,43], miR-155 was the most dramatically up-regulated in classically activated M1(LPS + IFN-γ) but not alternatively activated M2(IL-4) conditions. These results corroborate recent findings, which showed that miR-155 was differentially expressed in murine [45] and human [46,47] M1 and M2 macrophages. Although it is known that miR-155 is important in regulating inflammation [48], its key function in regulating distinct macrophage effector cells is novel. Other miRNAs, such as miR-27a, miR-29b, miR-125a, miR-146a, miR-122, miR-181a, miR-204-5p and miR-451 are differentially up-regulated in M1 and M2 spectrum macrophages [45,46]. However, miR-155 is unique in that it is very quickly (within 6 hours) and robustly (around a 100–180 FC increase) up-regulated during M1 differentiation. Elegant studies by O’Connell *et al* have shown that microbial and pro-inflammatory stimuli independently promote miR-155 [49]. For example, IFN-γ or TNF-α were shown to independently up-regulate miR-155 in the absence of LPS stimulus [49]. Overall, miR-155 is downstream of many inflammatory stimuli via NF-kB and other pathways [49]. The convergence of multiple inflammatory pathways into miR-155 expression highlights the importance of what are the consequences of miR-155 expression on the inflammatory phenotype of macrophages. Additional work will be required to exactly quantify IFN-γ's synergistic role in miR-155 up-regulation and downstream effects on M1 phenotype. Whether the drastic up-regulation of miR-155 in M1 macrophages is necessary to globally suppress many direct targets or very efficiently suppress a few key targets remains to be determined. Our data show that miR-155 controls expression of ~51% of all genes that define the M1(LPS+IFN-γ) phenotype. This is consistent with reports that miR-155 is downstream of several molecules necessary for induction of the M1 phenotype, such as TLRs [50] or Akt2 activity [51]. Expression of M1 markers was also reduced with miR-155 oligonucleotide inhibitors, suggesting a central role of miR-155 in establishing the M1 phenotype. Overall, these data point to a large and central role of miR-155 in regulating M1 phenotype.

It is still not known how miR-155 regulates inflammatory phenotype but miRNAs generally suppress gene expression. In WT M1(LPS + IFN-γ) macrophages–in which miR-155 is strongly induced–we identified 370 predicted or proven miR-155 target genes that are decreased to some extent. This suggests that the typical M1(LPS + IFN-γ) phenotype requires direct repression of hundreds of genes by miR-155. Among the most repressed miR-155 targets we confirmed we found *Inpp5d*, *Tspan14*, *Ptprj* and *MafB* [52–55]. We hypothesize that suppression of these genes is required for enhancement of M1-promoting pathways such as Akt2 [51] and Notch1 signaling [56,57], as well as inflammatory cytokine production. Supporting this, loss of *Inpp5d*/SHIP-1 in macrophages promotes Akt signaling [58]. Ptprj loss also strongly promotes Akt signaling. Since Akt2 is known to be required for M1 polarization [51], it is possible that miR-155-mediated suppression of *Inpp5d*/S and Ptprj in M1(LPS + IFN-γ) macrophages promotes Akt2 signaling. Interestingly, a miR-155/Akt2 positive feedback loop may exist, as Akt2 is required for complete miR-155 up-regulation [51]. In Akt2 KO mice, such lack of miR-155 decreased M1 and promoted M2 phenotype in a CEBPβ-dependent manner [51]. In contrast, we did not observe decreases in CEBPβ in M1macrophages. It is important to note that loss of *Inpp5d*/SHIP-1 had previously been found to be required for M2 differentiation [59] and we confirmed decreases in *Inpp5d* transcripts occur in both M1 and M2 cells, indicating that this is a common pathway in activated macrophages. Maf, an experimentally proven miR-155 target [55], may mediate cytokine effects, as it directly represses IL-12 transcription and indirectly represses other inflammatory cytokines [60]. Accordingly, we observed a complete lack of expression of IL-12 in any conditions except for WT M1(LPS + IFN-γ) macrophages (S3 **Table**), suggesting this is an important link in inflammatory phenotype mediated by miR-155. Finally, the contribution of Tspan14, a transmembrane protein [61–63], may be mediated by its interactions with ADAM10, which modulates M1-promoting Notch1 signaling [56,57]. Overall, our data support the hypothesis that inflammatory macrophage phenotype develops as a consequence of miR-155-dependent suppression of genes that inhibit the M1(LPS + IFN-γ) phenotype.

Interestingly, the down-regulation of several M2 markers that normally occurs during M1 differentiation was dampened in the KO M1(LPS + IFN-γ) macrophages, with a very strong effect on mannose receptor. The M2-exclusive marker Egr2 [17] was also less suppressed in miR-155 KO macrophages. However, the level of expression of these markers resembled more that of an M0 than an M2 macrophage, supporting that miR-155 is required for M1 differentiation and that its loss maintains macrophages in a more M0-like state.

The classical M1 and alternatively activated M2 phenotypes represent *in vitro*-derived extremes of a spectrum of *in vivo* macrophage phenotypes that change as a function of the inflammatory milieu [6,7,64]. Still, acute inflammatory macrophage responses *in vivo* express characteristics of *in vitro* M1 macrophages [17] and these cells are known to play a beneficial role in fighting infectious agents. However, excessive M1 responses can drive chronic inflammation leading to tissue injury or autoimmune disease [8]. In these scenarios, reducing M1 responses would be a therapeutically desirable option. Our data indicate that miR-155 plays a critical role in development of inflammatory M1(LPS + IFN-γ) responses, with particular emphasis on NO and IL-12 signaling pathways. Published data support that miR-155 plays a similar role in *in vivo* inflammatory disease models. miR-155 drives the inflammatory effects of TREM-1 in acute lung injury [65], mediates TNF-α, IL-1β and ROS in ischemia reperfusion injury [66] and promotes autoimmune lupus [67] and inflammation-induced neurological dysfunction [68]. The fact that our *in vitro* data shows that miR-155 oligonucleotide inhibitors are capable of producing effects similar to a miR-155 deficiency in macrophages provides promise for designing therapeutic strategies aimed at dampening inflammatory macrophage-mediated disease.

In conclusion, we have identified miR-155 as a small RNA that has a critical defining effect on the inflammatory M1 macrophage response. As a key molecule driving inflammatory macrophage phenotype, miR-155 shows potential as a therapeutic target in a myriad of inflammatory diseases. Conversely, it may be beneficial to enhance miR-155 activity to improve resistance to infections. Further work to develop drugs or delivery systems that specifically target miR-155 signaling in macrophages will help translate these promising findings into new effective therapies.

## Supporting Information

S1 Fig Myeloid cell populations in lymphoid tissues in miR-155 knockout mice. Percentage of polymorphonuclear leuokocytes (PMNs: Ly6C^+^Ly6G^+^), CD11c^+^ dendritic cells (Ly6C^-^Ly6G^-^ CD11c^hi^), monocytes (Ly6G^-^CD11c^-^ CD11b^+^Ly6C^hi^) and macrophages (Ly6G^-^ CD11c^-^ CD11b^hi^ Ly6C^int^) determined using flow cytometry in (A) bone marrow, (B) lymph nodes and (C) spleen in wild-type (WT, n = 3) and knockout (KO, n = 3) mice. Data from one experiment representative of 2–3 independent experiments.
(TIF)

S1 Table Genes increased more than 2-fold in KO M1 vs. KO M0 macrophages. (XLSX)

S2 Table Genes decreased more than 2-fold in KO M1 vs. KO M0 macrophages. (XLSX)

S3 Table M1 macrophage markers compared between WT M1vs. WT M0 and KO M1 vs. KO M0 macrophages. (XLSX)

S4 Table M2 macrophage markers compared between WT M1vs. WT M0 and KO M1 vs. KO M0 macrophages. (XLSX)

S5 Table miR-155 targets down-regulated in WT M1 vs. WT M0. (XLSX)
