# Supplementary figures and images for: Control of the Inflammatory Macrophage Transcriptional Signature by miR-155
Source: PLoS One. 2016 Jul 22;11(7):e0159724. doi: 10.1371/journal.pone.0159724 (PMC4957803; doi:10.1371/journal.pone.0159724)

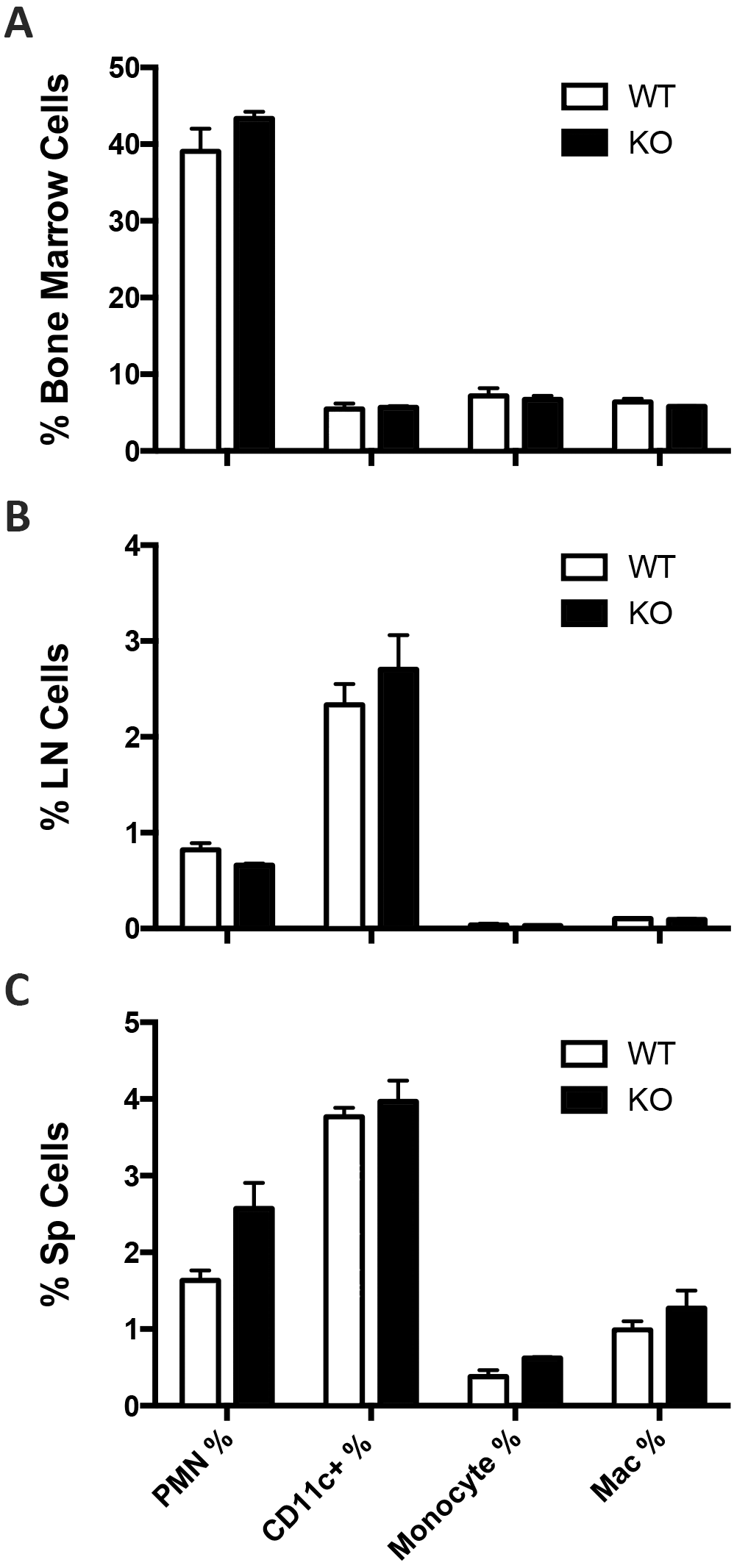

Supplement: S1 Fig — Percentage of polymorphonuclear leuokocytes (PMNs: Ly6C+Ly6G+), CD11c+ dendritic cells (Ly6C-Ly6G- CD11chi), monocytes (Ly6G-CD11c- CD11b+Ly6Chi) and macrophages (Ly6G- CD11c- CD11bhi Ly6Cint) determined using flow cytometry in (A) bone marrow, (B) lymph nodes and (C) spleen in wild-type (WT, n = 3) and knockout (KO, n = 3) mice. Data from one experiment representative of 2–3 independent experiments. (TIF) [file pone.0159724.s001.tif]
